# Subtle imaging signs of sigmoid sinus thrombosis in otitis media (“otitic hydrocephalus”)

**DOI:** 10.1016/j.radcr.2023.06.041

**Published:** 2023-06-28

**Authors:** Alejandra M. Maiz, Emily Chang, Tatiana K. Deveney, John Kim, Jonathan D. Trobe

**Affiliations:** a Department of Ophthalmology and Visual Sciences, University of Michigan, Ann Arbor, MI; b Department of Radiology (Neuroradiology), University of Michigan, Ann Arbor, MI; c Department of Neurology, University of Michigan, Ann Arbor, MI; d Kellogg Eye Center, 1000 Wall St, Ann Arbor, MI 48105, USA

**Keywords:** Otitic hydrocephalus, Papilledema, Mastoiditis, Dural venous sinus thrombosis

## Abstract

A 3-year-old boy developed otitis media, mastoiditis, papilledema, sixth nerve palsy, and increased intracranial pressure. The initial diagnosis was idiopathic intracranial hypertension, but doubt about that diagnosis at such a young age led to imaging reevaluation. When the abnormalities from multiple pulse sequences were aggregated with this clinical input, the correct diagnosis of otitic hydrocephalus emerged, allowing prompt implementation of appropriate treatment to avoid the risk of venous stroke.

## Introduction

The combination of otitis media, mastoiditis, papilledema, and increased intracranial pressure (ICP) with a bland cerebrospinal fluid (CSF) is known as “otitic hydrocephalus” [1]. Headache, fever, and unilateral or bilateral sixth nerve palsy are additional attributes. This condition is now understood to be precipitated by dural venous sinus thrombosis (DVST) resulting from spread of infection from the mastoid region to the sigmoid sinus. This case illustrates that the imaging signs of DVST causing otitic hydrocephalus can be extremely subtle, requiring an aggregation of multiple pulse sequences on magnetic resonance imaging (MRI) [2].

## Case report

A 3-year-old boy presented to an urgent care center with bilateral ear pain of several days’ duration. He received a diagnosis of bilateral otitis media and treatment with oral cefidinir 250 mg daily.

Despite the treatment, ear pain gave rise to holocranial pain. He began slapping his head with his hands and screaming that he was “going to die.” He also reported double vision and his parents noted that the left eye had turned inward. An ophthalmologist found papilledema and a left sixth nerve palsy and an otolaryngologist found inferior dullness of both tympanic membranes.

Brain magnetic resonance imaging (MRI) was interpreted as showing nonspecific signs of increased ICP, including widened optic nerve sheaths, a flattened pituitary gland, and narrowing of the transverse-sigmoid venous sinus junctions. Lumbar puncture (LP) disclosed an elevated opening pressure of 44 cm H_2_O and normal CSF constituents. With this imaging interpretation, the diagnosis was idiopathic intracranial hypertension (IIH), for which he was treated with an elixir of acetazolamide 375 mg/day.

**Fig. 1:**
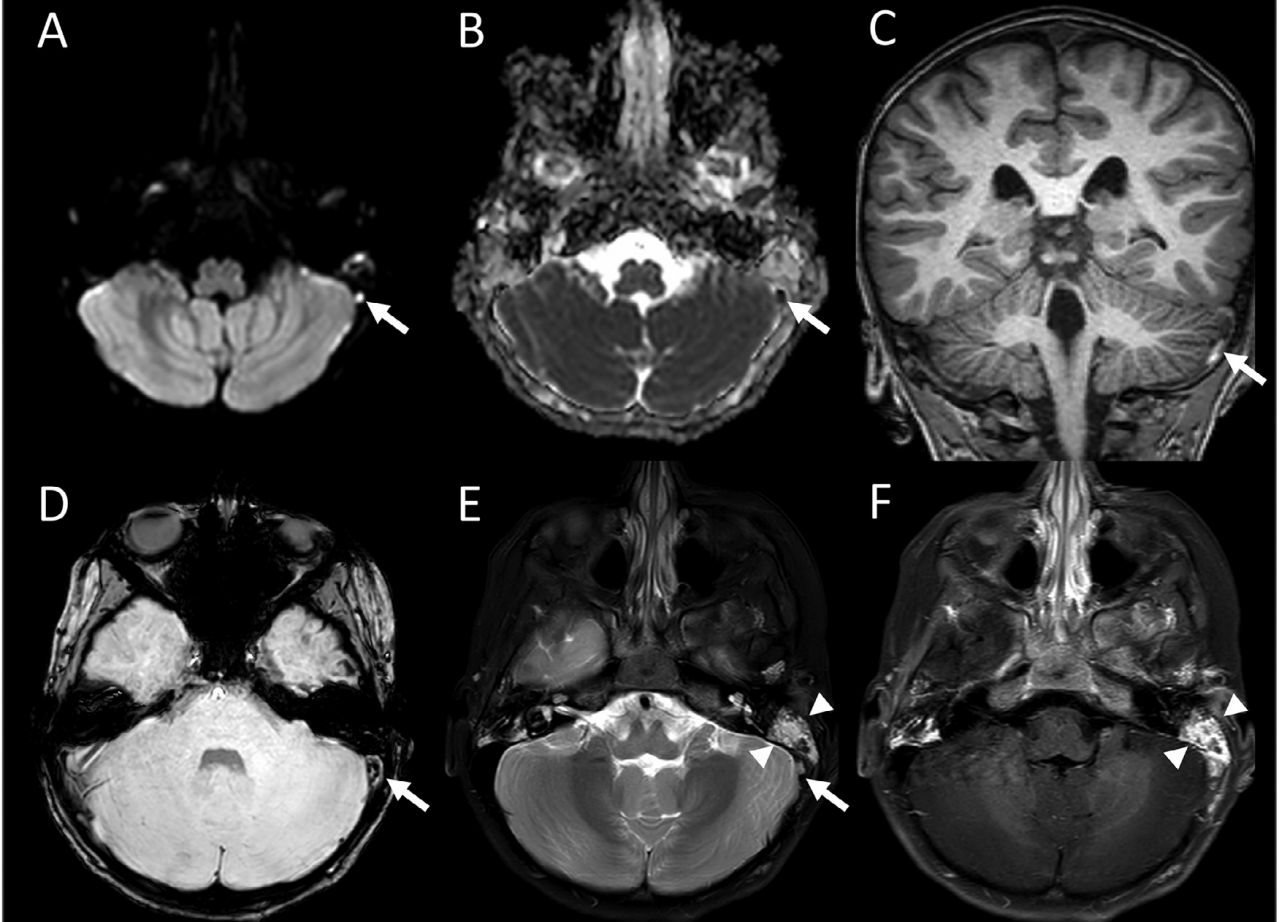
Magnetic resonance imaging (MRI). Axial diffusion-weighted imaging (DWI) (A) and corresponding apparent diffusion coefficient (ADC) map (B) demonstrate a focus of restricted diffusion in the left sigmoid sinus (white arrows), consistent with intraluminal thrombus. Coronal precontrast T1-weighted image (C) demonstrates intrinsic T1 shortening (white arrow) caused by methemoglobin, the oxidized hemoglobin of a subacute thrombus. Axial susceptibility-weighted imaging (D), which is extremely sensitive to iron-containing blood breakdown products, shows a dark signal (“blooming”), indicative of thrombus (arrow). Axial T2-weighted high intensity signal (E) indicates left mastoid fluid (arrowheads). Loss of the normal dark flow void in the adjacent left sigmoid sinus (arrow) indicates thrombosis. Axial postcontrast T1-weighted image shows enhancement (F) caused by mastoid inflammation (arrowheads).

**Fig. 2:**
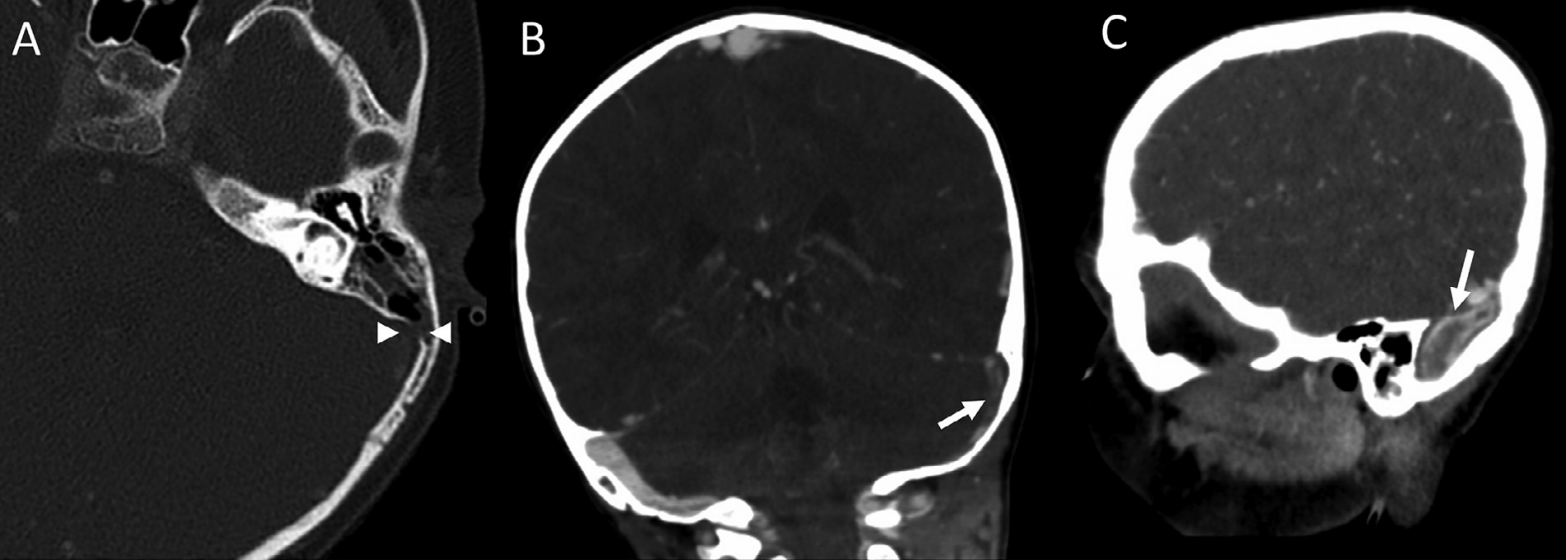
Computed tomographic venography (CTV). Axial (A) image demonstrates marked thinning of the left sigmoid sinus bony plate (arrowheads) caused by chronic mastoiditis. Coronal (B) and sagittal (C) images demonstrate iodinated contrast flowing around the thrombus, consistent with an incompletely occluded sigmoid sinus (arrows).

However, as IIH is exceedingly rare in early childhood, imaging reevaluation was requested. Review yielded the following abnormalities (Figs. 1 and 2): 1) T2-weighted hyperintensity and postcontrast T1-weighted enhancement in the left mastoid region, indicative of mastoiditis; and 2) left sigmoid sinus loss of normal flow void, restricted diffusion, increased susceptibility (“blooming”), and precontrast T1-weighted shortening, indicative of an intraluminal blood clot. An immediate computed tomography venography (CTV) then confirmed the DVST, based on evidence of a filling defect in the left sigmoid sinus and erosion of the left sigmoid sinus plate.

A hypercoagulability evaluation was normal. The patient underwent left mastoidectomy and a 10-day course of amoxicillin-clavulanic acid. He was placed on intravenous heparin and then transitioned to subcutaneous enoxaparin for a 12-week course. The acetazolamide treatment was continued for 3 months. At hospital discharge, he was afebrile and in good spirits. Examination one month after discharge was entirely normal.

## Discussion

Although the term “otitic hydrocephalus” was introduced in 1931 [3], the mechanism of the increased ICP was in doubt in the preimaging era. In fact, otitic hydrocephalus was often misapplied to patients with a condition now known as IIH [1]. In some such cases, however, Symonds [3] did note lateral (transverse) DVST at the time of surgery and proposed that the increased ICP resulted from “thrombophlebitis” with retrograde extension into proximal dural venous sinuses.

The incidence of DSVT in otitis media is low. Among 100 patients with “CNS complications” of otitis media, Gower and McGuirt identified only 5 cases of otitic hydrocephalus [4]. Investigators have posited that a high-riding jugular bulb with dehiscence of overlying temporal bone is a risk factor, such that the dural venous sinus becomes close to the middle ear, promoting spread of inflammation and activation of clot-forming factors [5].

Although the DVST in our patient had not, by imaging criteria, extended retrograde to involve the more proximal dural venous system on either side of the brain, such a limited site of occlusion can impede CSF drainage enough to raise ICP [6]. In other reported cases with thrombosis limited to one sigmoid sinus, there has been no evidence for an alternative mechanism of raised ICP.

The diagnosis of otitic hydrocephalus may be challenging. There is no ventriculomegaly; mastoid region imaging abnormalities are so common in childhood that they may be dismissed as noncontributory; and the DVST may be limited to a small region. Correct imaging diagnosis depends on drawing subtle abnormalities from a combination of T1-weighted, T2-weighted, diffusion-weighted, and susceptibility-weighted MRI, together with MRV or CTV [7]. CT with bone windowing is also useful, as DVST may occur when infection produces erosion in the bordering mastoid temporal bone [5]. Such bone erosion probably occurs only with chronic mastoiditis, and indeed, some cases of otitic hydrocephalus have occurred without fulminant temporal bone disease [3].

If the DVST is overlooked on imaging, the patient may be misdiagnosed as having IIH, as in this case, and treated inappropriately. As a consequence, venous stroke or permanent loss of vision from papilledema may occur [8].

Treatment involves a prolonged course of antibiotics, often with mastoidectomy or tympanomastoidectomy. The role of anticoagulation to prevent clot propagation is controversial. Its justification is the safety of intravenous and subcutaneous low molecular weight heparin in children and the risk of stroke and death following cortical vein thrombosis [9,10]. However, a retrospective review disclosed a low likelihood of clot propagation without treatment [11]. To protect against vision loss from persistent papilledema, the high ICP must be treated with an agent such as acetazolamide to lower CSF production.

## Conclusion

The diagnosis of otitic hydrocephalus depends on identifying imaging signs of DVST, which may be subtle, requiring an aggregation of multiple MRI pulse sequences. Delay in diagnosis can result in retrograde spread of clot, leading to stroke and even death. Treatment consists of a combination of systemic antibiotics, tympanomastoidectomy, anticoagulation, and ICP-lowering medication. To be effective, it must be instituted early.

## Patient consent

The patient's family gave written permission for the publication of the facts and images of this case.
